# Efficacy of an online cognitive behavioral therapy program developed for healthcare workers during the COVID-19 pandemic: the REduction of STress (REST) study protocol for a randomized controlled trial

**DOI:** 10.1186/s13063-020-04772-7

**Published:** 2020-10-21

**Authors:** Luisa Weiner, Fabrice Berna, Nathalie Nourry, François Severac, Pierre Vidailhet, Amaury C. Mengin

**Affiliations:** 1 Pôle de Psychiatrie, Santé Mentale et Addictologie, Hôpitaux Universitaires de Strasbourg, 1 place de l’hôpital, 67000 Strasbourg, France; 2 Laboratoire de Psychologie des Cognitions, Université de Strasbourg, 12 rue goethe, 67000 Strasbourg, France; 3 INSERM U1114 Neuropsychologie Cognitive et Physiopathologie de la Schizophrénie, Strasbourg, France; 4 Faculté de Médecine, Université de Strasbourg, Strasbourg, France; 5 Service de Pathologies Professionnelles et Médecine du Travail, Hôpitaux Universitaires de Strasbourg, Strasbourg, France; 6 Département de Santé Publique, GMRC, Hôpitaux Universitaires de Strasbourg, Strasbourg, France; 7 Laboratoire de Biostatistique et Informatique Médicale, iCUBE UMR 7357, Université de Strasbourg, Illkirch, France; 8 Centre Régional Psychotraumatisme Grand Est, Strasbourg, France

**Keywords:** COVID-19, Randomized controlled trial, Protocol, Online CBT, Healthcare workers, Stress, Resilience, PTSD, Depression

## Abstract

**Background:**

The acknowledgment of the mental health toll of the COVID-19 epidemic in healthcare workers has increased considerably as the disease evolved into a pandemic status. Indeed, high prevalence rates of depression, sleep disorders, and post-traumatic stress disorder (PTSD) have been reported in Chinese healthcare workers during the epidemic peak. Symptoms of psychological distress are expected to be long-lasting and have a systemic impact on healthcare systems, warranting the need for evidence-based psychological treatments aiming at relieving immediate stress and preventing the onset of psychological disorders in this population. In the current COVID-19 context, internet-based interventions have the potential to circumvent the pitfalls of face-to-face formats and provide the flexibility required to facilitate accessibility to healthcare workers. Online cognitive behavioral therapy (CBT) in particular has proved to be effective in treating and preventing a number of stress-related disorders in populations other than healthcare workers. The aim of our randomized controlled trial study protocol is to evaluate the efficacy of the ‘My Health too’ CBT program—a program we have developed for healthcare workers facing the pandemic—on immediate perceived stress and on the emergence of psychiatric disorders at 3- and 6-month follow-up compared to an active control group (i.e., bibliotherapy).

**Methods:**

Powered for superiority testing, this six-site open trial involves the random assignment of 120 healthcare workers with stress levels > 16 on the Perceived Stress Scale (PSS-10) to either the 7-session online CBT program or bibliotherapy. The primary outcome is the decrease of PSS-10 scores at 8 weeks. Secondary outcomes include depression, insomnia, and PTSD symptoms; self-reported resilience and rumination; and credibility and satisfaction. Assessments are scheduled at pretreatment, mid-treatment (at 4 weeks), end of active treatment (at 8 weeks), and at 3-month and 6-month follow-up.

**Discussion:**

This is the first study assessing the efficacy and the acceptability of a brief online CBT program specifically developed for healthcare workers. Given the potential short- and long-term consequences of the COVID-19 pandemic on healthcare workers’ mental health, but also on healthcare systems, our findings can significantly impact clinical practice and management of the ongoing, and probably long-lasting, health crisis.

**Trial registration:**

ClinicalTrials.gov NCT04362358, registered on April 24, 2020.

## Background

The acknowledgment of the mental health toll of the COVID-19 epidemic both in the general population [1–5] and in healthcare workers [6–11] has increased dramatically in the last few weeks as the disease has grown into a pandemic status.

**Fig. 1:**
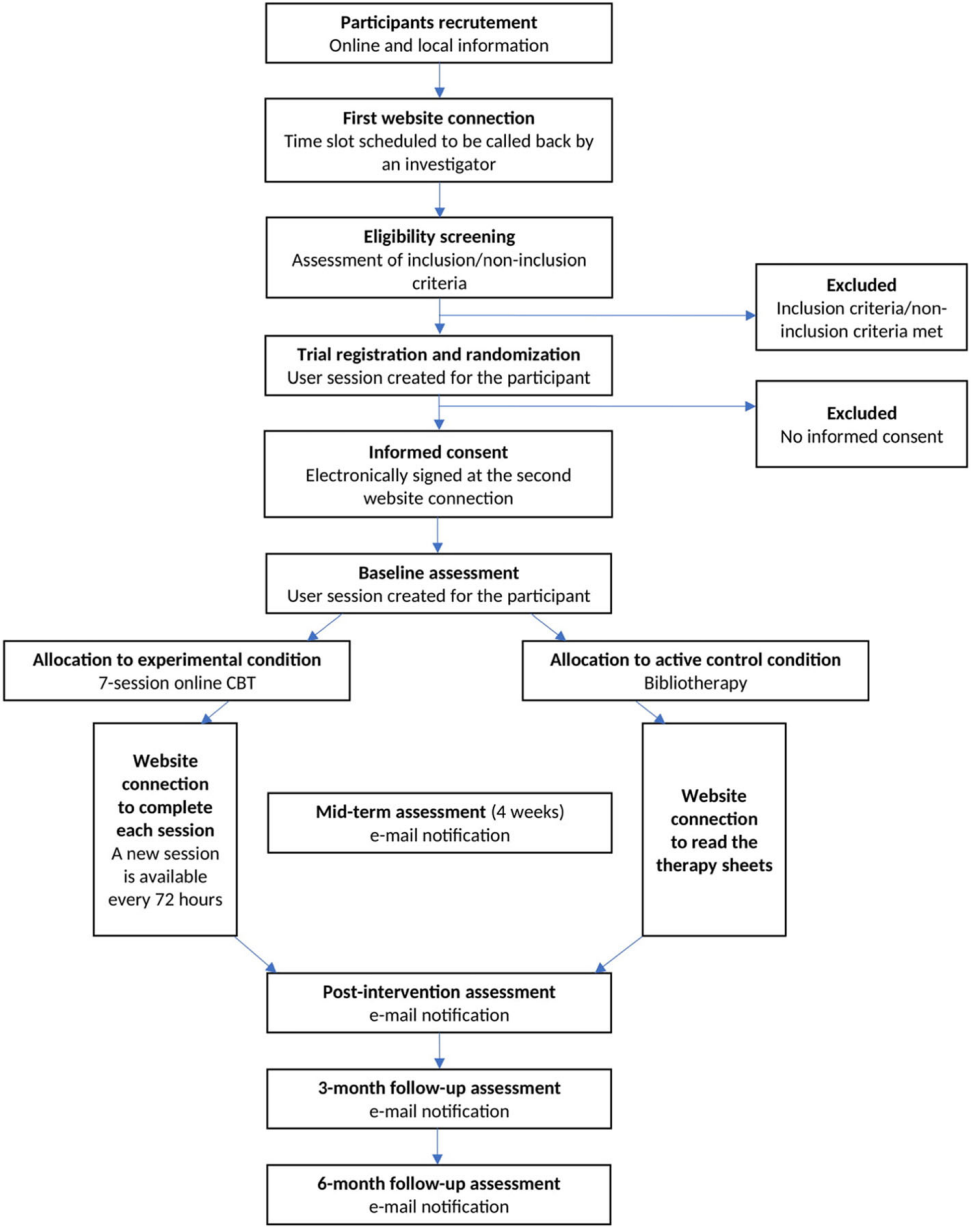
Flow chart of the REST study

Indeed, preliminary epidemiological and qualitative findings suggest that the epidemic might have negative immediate as well as long-term mental health effects, particularly in healthcare workers [12]. For instance, in one of the first reports published by a Chinese team, frontline healthcare workers were found to present with extremely high rates of depression (> 50%), generalized anxiety disorder (> 44%), insomnia (> 36%), and stress-related symptoms (> 73%) [13]. Since then, numerous studies have further confirmed these findings in nurses [11, 14], physicians and medical staff [6, 8, 12, 15], and trainees [10, 16] and warranted the need for psychological treatments aiming at relieving immediate stress and preventing the onset of psychological disorders in healthcare workers [12]. To address this need, we developed a 7-session online cognitive behavioral therapy (CBT) program targeting the reduction of stress and the reinforcement of adaptive coping behaviors in healthcare workers during the COVID-19 pandemic. The aim of our randomized controlled trial study protocol is to evaluate the efficacy of the treatment we have designed for healthcare workers—the ‘My Health too’ CBT program—on immediate perceived stress, as well as on the emergence of psychiatric disorders at 3- and 6-month follow-up (Fig. 1).

While the long-term mental health effects of the COVID-19 pandemic are still unknown, studies conducted following the SARS-CoV-1 outbreak in 2003 suggest that healthcare workers are particularly at risk of developing severe mental health disorders following the acute stages of the current pandemic [17, 18]. For instance, healthcare workers in hospitals in Toronto who treated SARS-CoV-1 patients continued to show symptoms of severe psychological distress 1 and 2 years post-outbreak [19, 20], with 13.8% presenting with significant symptoms of posttraumatic stress disorder (PTSD) [17]. Irrespective of the contamination status, fewer years of experience and personal history of psychiatric disorder were among the main predisposing factors involved in the emergence of psychiatric disorders [20]. In addition to effects on their individual well-being, there was also a long-term systemic impact of these mental health problems, as healthcare workers reported reduced patient contact and hours of healthcare work as well as more frequent sick absences and increased behaviors that could affect their work [19]. It is noteworthy that although the lethality of the SARS-CoV-2 is lower than that associated with the SARS-CoV-1, the SARS-CoV-2 is more contagious, and the COVID-19 has become much more widespread [14]. Hence, it is possible that the mental health impact of the current pandemic on healthcare workers, as well as its systemic impacts, will be equally or even more long-lasting and severe than those reported following the SARS-CoV-1 outbreak [12, 17].

A number of solutions were implemented in China to prevent psychiatric disorders in healthcare workers, such as telephone hotlines and face-to-face group or individual psychotherapy sessions [21]. However, healthcare workers’ use of these interventions was limited, probably due to workload as well as fear of contamination [21]. In this context, web-based programs represent a more feasible format, because of their high dissemination potential and flexibility. Indeed, such programs can be made available to a high number of people, who have variable schedules, an important workload, and who are afraid of being contaminated themselves in face-to-face intervention formats [13] (Table 1).

**Table 1:**
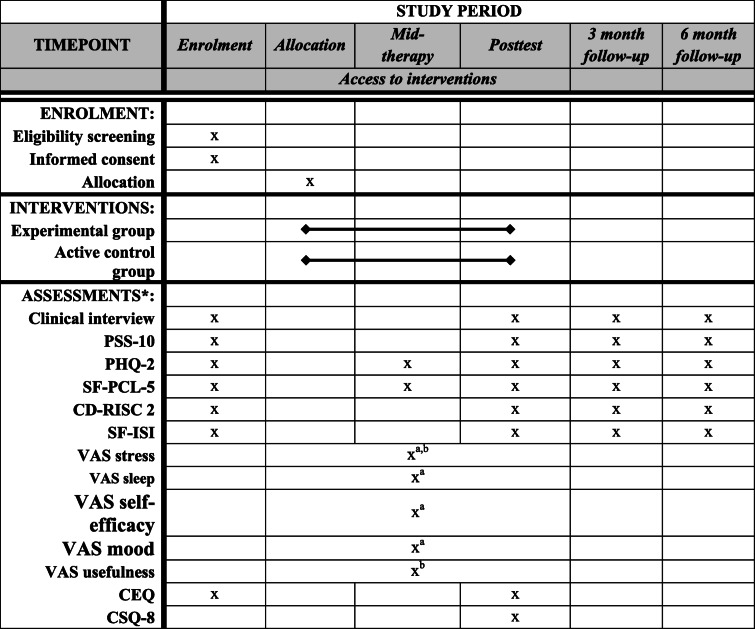
Schedule for the REST study

*PSS-10 Perceived Stress Scale, *PHQ-2* Patient Health Questionnaire, *SF-PCL-5* Posttraumatic stress disorder CheckList scale Short Form, *CD-RISC 2* Connor-Davidson Resilience Scale, *SF-ISI* Insomnia Severity Index Short Form, *VAS* visual analogic scale, *CEQ* Credibility Expectancy Questionnaire, *CSQ-8* Client Satisfaction Questionnaire

^a^Assessed in experimental group before each session

^b^Assessed in experimental group after each session

CBT has been found to be effective in the prevention of burnout in healthcare workers, in stressful contexts other than the current COVID-19 pandemic [22]. Moreover, there is evidence of the effectiveness of CBT in the prevention of a number of psychiatric disorders in at-risk individuals, such as PTSD [23] and depression [24]. Despite a growing interest in the field of web-based psychological interventions, internet-based CBT remains under-developed [24, 25], and, to our knowledge, internet-based CBT programs specifically designed to address stress-related conditions in healthcare workers are lacking [26].

Given its demonstrated efficacy and feasibility in the treatment of stress and in the cultivation of resilience in other populations (e.g., college students and firefighters) [25, 27, 28], it is crucial to fill this important gap in the research through the investigation of the efficacy of internet-based CBT programs targeting stress and resilience in healthcare workers. This is all the more urgent in the current pandemic context, which requires a number of unplanned adaptations of psychological interventions in order to address the psychological needs of patients [7], i.e., social distancing measures, working under extraordinary stressful circumstances, increased workload, and variable work schedules [21].

The aim of the present randomized controlled trial is to investigate the efficacy and the acceptability of a 7-session internet-based CBT program we have developed to address the immediate stress and prevent its long-term consequences, in healthcare workers during the COVID-19 pandemic. To assess its impact on perceived stress, we will compare the effects of the internet-based ‘My Health too’ 7-session CBT intervention to that of bibliotherapy on self-reported measures of perceived stress immediately following the 8-week intervention as well as at 3- and 6-months follow-up. We chose bibliotherapy as an active control comparison group because it offers participants unguided access to online self-help stress-reduction material, akin to ecological self-help situations, that should nevertheless be less efficacious than online CBT [29]. In both arms, human support is available to all participants included in the trial via their optional use of the COVID-19 psychological hotlines. Subjects will be randomly allocated, following a 1:1 ratio with stratification by site, either to the online CBT group or the bibliotherapy group. We expect scores on the 10-item Perceived Stress Scale [30] to be significantly decreased in the online CBT group compared to the bibliotherapy group over the course of the 8-week period of treatment. Moreover, participants in the online CBT group should present with decreased symptoms of PTSD, insomnia, and depression compared to the bibliotherapy group both immediately following the 8-week treatment period, but also at 3- and 6-month follow-up. In order to evaluate acceptability, we will assess attrition rates, client satisfaction, and therapy credibility. Finally, we were also interested in the psychological and contextual dimensions (e.g., medication, sleep quality, perceived resilience, rumination, post-traumatic, and depressive symptoms) that might mediate the efficacy of online CBT as well as the demographic, occupational, and medical data (e.g., experience on the job and psychiatric history) that might predict efficacy. This is crucial in order to improve treatment effectiveness in the future and the prevention of psychological disorders in healthcare workers.

## Methods/design

### Design

Our project consists of a six-site, prospective, randomized, open and parallel group-controlled study with two arms: an experimental arm with 7 online CBT sessions and an active control arm consisting of online bibliotherapy. In addition to these interventions, each participant enrolled in the study will be informed that they can make use of the psychological hotline via the website throughout the 8-week treatment duration, in case they need to have access to a qualified therapist. The study was approved by the relevant local ethics committee (Comité de Protection des Personnes Ile-de-France VI, May 7, 2020, N 36-20) and registered at ClinicalTrials.gov on 24 March 2020 (study identifier NCT04362358).

### Study population

Participants will meet the following inclusion criteria: (1) belong to one of the following professions: medical doctors, nurses, orderlies, physiotherapists, psychologists, hospital porters, ambulance drivers, nursing and medical students working in hospitals; (2) aged 18–70 years old; and (3) fluent in the French language. Exclusion criteria include (1) initial score at the Perceived Stress Scale-10 (PSS-10) [30]< 16 (which means that the participant has a non-significant level of stress), (2) suicidal ideation score assessed by the question 9 of the Patient Health Questionnaire - 9 (PHQ-9) [31] > 2, and (3) be under guardianship. Participants will be recruited by psychiatrists or psychologists in six hospitals of the East region of France, i.e., Hôpitaux Universitaires de Strasbourg, Hôpitaux Civils de Colmar, Groupe Hospitalier Régional de Mulhouse Sud-Alsace, Centre Hospitalier Universitaire de Nancy, Centre Hospitalier Universitaire de Besançon, and Centre Hospitalier Universitaire de Dijon; the East region was the region that was the most affected by the COVID-19 in France. Demographic information (gender, age, family situation), medical history, and occupational exposure (i.e., position, service, seniority on the job, working time, COVID-19 infection status) will be collected.

### Trial status

This is the first version of the protocol (May 7, 2020). Study enrollment has not started yet and will begin in June 2020. The active treatment phase is expected to be finished in September 2020. The completion of follow-up assessments is expected in November 2020 and February 2021. The total sample of 120 participants will be completed by February 2021.

### Sample size

The number of subjects required was calculated based on the data from Rose et al. [25] which showed a relative decrease in the PSS-10 score of 15% in the control group and 30% in the experimental group. Mean score values with a standard deviation (< 5) were reported. Given that the healthcare professionals recruited in our study will be under greater stressors than Rose et al.’s [25] study sample, which consisted of college students, the expected values of PSS-10 at baseline are 23 ± 5 with a decrease to 19.6 ± 5 in the control group and 16 ± 5 in the experimental group. Simulations using a mixed linear regression model showed that 46 subjects per group, i.e., 92 in total, would be needed to highlight a difference between groups with a power of more than 90% and an alpha risk set at 5%. Considering an estimation of 10% of exclusions (initial PSS-10 < 16) and 20% of drop-outs, the number of subjects in the study was increased to 120 subjects in total (Fig. 1).

### Outcomes

#### Primary outcomes

The decrease of the Perceived Stress Scale – 10 items version (PSS-10) [30] score at 8 weeks will be our primary outcome. The 10 items of the PSS range from 0 (never) to 4 (very often); higher scores are indicative of higher perceived stress. The PSS-10 is validated in French [32], and its ease of use and psychometric properties have been widely studied in professional contexts [33]. The expected PSS-10 values are 23 ± 5 initially with a decrease to 19.6 (control group) and 16 (experimental group).

#### Secondary outcomes

The Patient Health Questionnaire – 2 items version (PHQ-2) [34] will be applied to screen for depression. Each item is scored on a scale ranging from 0 (not at all) to 3 (nearly every day).

The Short Form Posttraumatic Stress Disorder Checklist 5 (SF-PCL-5) [35] will be used to screen for post-traumatic stress symptoms. It consists of 4 items ranging from 0 (not at all) to 4 (extremely).

The Connor-Davidson Resilience Scale – 2 items version (CD-RISC 2) [36] will be used to measure resilience. The 2 items are scored on a scale ranging from 0 (not true at all) to 4 (almost always true).

A short form of the Insomnia Severity Index (ISI) [37] will be used to measure the severity of insomnia using 5 of the initial 7 items of the ISI. Each item is scored on a scale ranging from 0 (not at all) to 5 (extremely).

The Affective Rumination Questionnaire (ARQ) [38] will be applied to measure work-related rumination. The 5 items are rated on a scale ranging from 0 (very rarely or never) to 4 (very often or always).

Self-reported credibility of the treatment will be assessed via the Credibility and Expectancy Questionnaire (CEQ) [39] at baseline and at 8 weeks of treatment. This questionnaire contains 6 items rated either from 1 to 9 or from 0 to 100% and evaluates whether the participants “think” or “feel” that their treatment will be efficient.

Finally, the Client Satisfaction Questionnaire (CSQ-8) [40] will be applied as a self-report measure assessing participants’ satisfaction at the end of the 8-week program. The 8 items are scored on a scale ranging from 1 to 4. Higher scores are indicative of elevated satisfaction.

To measure the perceived efficacy and utility of each individual session of the CBT program, participants will complete visual analog scales (VAS) measuring stress, sleep, self-efficacy, and mood before each session; two VAS will be administered after each session to measure perceived stress and utility of the session.

The following measures will be used to assess acceptability: the number of participants having completed the program (i.e., ≥ 5/7 sessions) compared to the number of participants connected to the platform (number of drop-outs); the program completion time (from minimum 3 weeks to maximum 8 weeks); the utility of each session, assessed via the VAS after each session; how often the telephone hotline was used; and over the course of the 7 sessions, the relative ratings of perceived stress and self-efficacy and how often subjects practiced the skills associated with each session (Table 1).

### Procedure

Participants will be recruited via (i) the psychological support hotlines established during the COVID-19 outbreak at the 6 hospital sites participating in the study, (ii) the psychologists and psychiatrists who consult with healthcare workers in their departments, (iii) by billposting, and (iv) by posting on the hospital websites and on targeted Facebook groups. An e-mail will be sent to department heads to inform their team members. Subjects interested in taking part in the study will be asked to consult the study website (www.masanteaussi.fr) where they will find a document with information on the aims and the scope of the study. They will then choose a time slot to be called back by an investigator who will assess the subject’s eligibility. Oral information about the study and the randomization procedure will be given to all subjects. Participants will be randomized and assigned to the intervention by the investigator. If the subject is eligible, he/she will receive an e-mail with a username and password allowing him/her to log into the platform after a 24-h period. After receiving the e-mail, participants will be able to make their first connection to the website and sign an electronic informed consent form (previously signed online by the investigator). They will then have to complete the self-report questionnaires assessing symptoms of depression, PTSD, insomnia, resilience, and rumination. Following this, they can either start their first session of the online CBT program or gain access to the bibliotherapy documents.

Their active participation in the program will last 8 weeks. Throughout the 8-week duration of the program, each participant (either in the experimental or the control condition) will have access to the psychological hotline (from Monday to Friday between 9 a.m. and 5 p.m.), if they need support in using the resources made available to them. We chose to provide supported interventions instead of fully automated, stand-alone internet-based CBT and bibliotherapy, as the latter generally have higher treatment dropout rates (74%) than those provided with therapist or administrative support (28% and 38%, respectively) [41]. Moreover, in the context of the current pandemic and its social distancing-related guidelines, actual social support has been highlighted as particularly necessary in order to improve well-being [42]. During the study, participants are allowed to benefit from any medical or psychological follow-up or to take psychotropic drugs when prescribed by a medical doctor.

Mid-therapy assessment will take place at 4 weeks after inclusion, i.e., subjects will be asked to fill out depression, PTSD, and COVID-19 infection status questionnaires in order to track potential undesirable outcomes. Eight weeks following their inclusion, participants will receive an e-mail inviting them to fill out all the self-report questionnaires. Then, an e-mail will inform them of the end of the program and of the availability of local mental health services if needed, e.g., psychologists, psychiatrists. Three and 6 months after the end of the program, participants will receive an e-mail inviting them to fill out the follow-up self-report questionnaires. At each step (4-week, 8-week, 3-, and 6-month follow-up), undesirable outcomes will be tracked: i.e., when a participant presents with significant suicidal ideation scores on the 9th item of the PHQ-9 (> 1), the investigator is automatically notified by the website and calls the participant to make the appropriate referrals (e.g., psychiatrist); if a participant is hospitalized in a psychiatric facility during the 8-week treatment phase, he or she will be removed from the study. If a questionnaire is not completed 3 days after it is due, the participant will receive a reminder via an e-mail notification. If questionnaires are not completed a week after they are due, one of the investigators will call the participant and invite him/her to complete it. At any time during the intervention period and after completing the program, subjects are free to withdraw their participation without justifying their motives. No further data will be collected for these participants.

The coordinating center will be led by LW, ACM, and NN and include 5 additional psychologists. Their role is to supervise the inclusion process (communication, information of potential recruits, consent) and to explain and guide the other centers in their own inclusion and assessment process, providing standardized documents for the inclusion call, communication means, and data storage. This coordinating group will meet weekly. A scientific committee composed of the main investigator (LW), two associated investigators (NN, ACM), a methodologist (FS), and a promoter representative wrote the protocol, selected the investigators, and decided whether to modify, continue, or stop the project. A data management team composed of two data managers configured the online survey software (*CleanWeb*) and made sure data storage was secure. Any modification of the protocol, of the instructions or of the consent form will be referred to the ethics committee (Comité de Protection des Personnes Ile-de-France VI). Modified and approved versions of these documents will be transmitted by the promoter to the study coordinating team and to the French agency for health products and medications safety (Agence Nationale de Sécurité du Médicament et des produits de santé, ANSM). The promoter will update the information about the trial on Clinicaltrials.gov.

### CBT intervention: my health too

We developed the CBT program “My Health Too” that consists of 7 video sessions of approximately 20 min, which target the following components identified as key to increasing resilience to stress and preventing mental health problems [27, 28]: (i) psychoeducation [43], (ii) functional behavioral and cognitive coping strategies [43], (iii) mindfulness, (iv) mindfulness/acceptance [44], (v) promoting action toward values [44], (vi) addressing barriers and motivation to use self-compassion as a psychological skill [45], and (vii) self-compassion to soothe difficult emotion [45, 46]. A new session is available every 72 h. Each session is preceded and followed by VAS to aid participants to identify their subjective manifestations of stress and assess the utility of the session. In addition, by the end of each session, participants have the possibility to call a psychologist from the hotline and are invited to practice the strategies learned between sessions through the use of the homework material associated with each video, including mindfulness and relaxation exercises (see Table 2 for the content of the sessions). Whenever a participant does not complete a given session 72 h following the first notification of its availability, he/she will receive an e-mail notification as a reminder. When a participant does not complete a given session 1 week after the first notification of its availability, he/she will be called by an investigator.

**Table 2:** Title and content of sessions of the CBT program My Health too

Session number	Session title	Session content
1	The psychological mechanisms of stress	Psychoeducation on Lazarus and Folkman’s [43] transactional stress model; cognitive restructuring
2	Useful behaviors during highly stressful situations	Functional coping strategies (e.g., problem-solving, social support, relaxation, and cognitive restructuring)
3	Mindfulness in everyday life	Mindfulness vs. automatic pilot; mindful observation; 3-min mindfulness practice; letting go of the automatic pilot to improve sleep
4	Dropping the anchor in the present moment to increase resilience	Mindfulness/acceptance skills (i.e., dropping the anchor, cognitive defusion)
5	Engaging in valued actions	Understanding how values are linked to emotions; identification of values, and valued actions
6	Self-compassion to improve self-care	Psychoeducation on compassion as a psychological skill to soothe difficult emotions and self-criticism [45]; barriers to self-compassion; compassion as a means to self-care
7	Self-compassion to improve emotion regulation	How to use self-compassion to soothe difficult emotions; using self-compassion as a means to self-care; compassion-focused mindfulness [46]

### Active control condition: bibliotherapy

Bibliotherapy consists here of brochures with self-help written relaxation material, which provides low-intensity intervention for stress that should be less efficacious than online CBT [29]. Participants will be able to download these brochures, in a pdf format, via the MaSanteAussi.fr website. The brochures contain psychoeducation as well as written and illustrated instructions to guide relaxation and mindfulness practices. In addition to the written material, people will also have the possibility to call the psychological hotline throughout the 8-week duration of the study.

### Hotline organization

A hotline specifically dedicated to the participants will be set up, run by a team of CBT-trained psychologists. The hotline will be available from Monday to Friday from 9 a.m. to 5 p.m. The psychologists will be trained on the bibliotherapy resources as well as on the CBT “*My Health Too*” online program (a half-day training will be given to them beforehand). They will provide psychological support, advice concerning the skills taught in the program (from either the CBT “My Health too” program or the bibliotherapy brochures), and CBT-based stress management techniques. If the participant presents with more severe psychological symptoms (i.e., severe depression or suicidal ideation), the psychologist will make the appropriate referrals (e.g., psychiatrist). In addition, weekly supervision will be provided by the senior psychologists who created the program. Outside of the hotline’s opening hours, an answering machine will inform participants that they can call the local psychiatric services in case of an emergency.

### Website

The “My Health Too” website was initially developed by a team of developers, designers, illustrators, and videographers during a Hacking Health Camp event—i.e., the HackingCovid-19 which took place from March 18, 2020, to April 10, 2020. The site will be hosted by the University Hospital of Strasbourg, accredited to host health data. All data collected will be anonymized, coded, and stored on this secure server. The management of the trial data will be carried out using the CLEANWEB® software solution marketed by TELEMEDICINE Technologies S.A.S. The collection and storage of data complies with the General Data Protection Regulation (GDPR). A table of correspondence between the names and anonymous identifiers will be kept by the investigators in paper format, in secure facilities at their center. The main investigators will have access to the final dataset (LW, FB, ACM). Any data required to support the protocol can be supplied on request.

### Randomization

Randomization will be carried out using the CLEANWEB® software. The randomization will be stratified by the investigation center with a 1:1 ratio for allocation to the 2 groups. Blocks of varying sizes will be randomly selected. The allocation sequence was configured by our data manager who is blinded to the allocation group. Participants will be informed of the group which they are allocated to.

### Statistical analyses

A descriptive analysis will be performed on the entire population and in each group (experimental and control). Categorical variables will be described, giving the numbers and frequency of each modality. Quantitative variables will be described using the usual positional and dispersion parameters. The data analyst will be blinded as he will not participate to data collection.

We will carry out the statistical analysis of the main judgment criterion using a mixed model, including a “group” effect (experimental or control), a time effect (J0 and S8), an interaction between the “group” and the “time,” and a “subjects” effect and a “center” effect. The “subject” and “center” effects will be random effects. The statistical test of interest will focus on the interaction term between group and time, which will assess whether the decrease in the PSS-10 score is greater in the experimental group than in the control group. In a second step, if this test is significant, we will estimate the decrease difference between the groups.

As of the analyses of secondary endpoints, we will assess the efficacy of online CBT on the prevention of PTSD, insomnia, and depression at 3 and 6 months using mixed regression models. In the case of restricted scores with values close to the extremes, we may use beta regression models after transformation of the variable of interest on a scale of ]0;1[. Regarding the predictive factors of the efficacy of online CBT, we will also use the aforementioned models, incorporating an interaction between group, time, and each potential predictive factor to test whether the improvement of scores in the CBT group relies on another factor. Also, we will carry out the identification of potential mediating factors explaining the effect of CBT on professional stress using Bayesian networks that allow learning the structure of a system from the data while integrating hypotheses about the structure. Mediation formulae will be used to assess total, direct and indirect effects. Finally, we will perform a descriptive analysis of the indicators of acceptability and satisfaction.

In order to minimize attrition bias, an intention to treat analysis and specific techniques for handling missing data will be performed. Missing data will be described variable by variable and as a whole in order to search for monotonous patterns. In order to determine the process of generating the missing data (MCAR: missing completely at random, MAR: missing at random or MNAR: missing not at random), dummy variables for whether a variable is missing will be created and cross-tabulated with others observed variables. Multivariate logistic regression can be used to determine a set of variables associated with the probability of observation. In the case of MCAR or MAR processes, multiple imputation methods will be used.

## Discussion

To the best of our knowledge, this will be the first randomized controlled trial comparing the efficacy and the acceptability of a brief online CBT program specifically developed for healthcare workers and of an active control group (i.e., bibliotherapy). Given the potential short- and long-term consequences of the COVID-19 pandemic on healthcare workers’ mental health, at an individual level, but also on healthcare systems, on a systemic level [11, 12, 17], our findings can significantly impact clinical practice and management of the ongoing, and probably long-lasting, health crisis [21].

Indeed, if our 7-session online CBT program produces a significant decrease on immediate perceived stress levels and improves the prevention of severe psychiatric disorders, such as PTSD and depression, it could become an empirically assessed viable option specifically suited to the needs of healthcare workers facing the COVID-19 pandemic and might be easily adapted to other sanitary crises. Indeed, in terms of format and content, this brief online CBT format has many advantages in the current context: first, similar programs have proved to improve resilience in highly stressful situations and prevent the emergence of psychological disorders [25]; second, it circumvents some of the pitfalls associated with face-to-face interventions (e.g., fear of contamination, and social distancing measures) and provides the flexibility required in order to facilitate its accessibility to a great number of healthcare workers [21]. In addition, we have chosen a step-by-step guided format rather than a free access to the program sessions in a random order. This format mimics the outline of standard face-to-face CBT possibly enhancing the effectiveness of the program. It will also allow us to assess the acceptability and adherence to such a format, as well as their links with participants’ use of the support from the hotline [41].

Our study protocol has some limitations. First of all, this study design does not include a control arm that could account for the effects of the passage of time. Although this could provide a more rigorous test of our study hypotheses, a third control arm (e.g., wait-list or treatment as usual) was ruled out due to ethical concerns. Second, though concomitant psychotropic medications as well as the use of adjunctive therapeutic means (e.g., self-help or counseling) will be controlled in our study, subjects will not be instructed to avoid making use of them. While this might be a potential confound in the current study design, the restriction of therapeutic options would also pose an ethical concern, reduce referrals to the study and also be a threat to external validity. Therefore, by monitoring therapeutic (i.e., medication or counseling) use throughout the trial, the current study seeks to balance the internal and external validity and improve the feasibility of the study while efficiently addressing its hypotheses. To summarize, if our study hypotheses are confirmed, our program has the potential to become the first evidence-based option developed for the treatment of stress-related conditions (i.e., the COVID-19 psychological burden) in healthcare workers.

### Study timeframe

Study enrollment will begin in June 2020, by the end of the epidemic peak in the East region of France. The active treatment phase is expected to be finished in September 2020. The completion of follow-up assessments is expected in November 2020 and February 2021. The total sample of 120 participants will be completed by February 2021. The dissemination of first results at international meetings is expected by October 2020. Publication of the findings is planned for March 2021.

## Supplementary information

**Additional file 1.** SPIRIT 2013 Checklist: Recommended items to address in a clinical trial protocol and related documents.

## Data Availability

The study protocol has been reported in accordance with the Standard Protocol Items: Recommendations for Clinical Interventional Trials (SPIRIT) guidelines (Additional file 1). The consent form and information material are available from the corresponding author on request.

## Abbreviations

ARQ: Affective Rumination Questionnaire
CBT: Cognitive behavioral therapy
CD-RISC-2: Connor-Davidson Resilience Scale – 2-item version
CEQ: Credibility and Expectancy Questionnaire
CSQ-8: Client Satisfaction Questionnaire – 8-item version
ISI: Insomnia Severity Index
PHQ-2: Patient Health Questionnaire – 2-item version
PSS-10: Perceived Stress Scale – 10-item version
PTSD: Post-traumatic stress disorder
SF-PCL-5: Short Form Posttraumatic Stress Disorder Checklist
VAS: Visual analog scale

## References

[1] Cao W, Fang Z, Hou G, Han M, Xu X, Dong J (2020). The psychological impact of the COVID-19 epidemic on college students in China. Psychiatry Res.

[2] Duan L, Zhu G (2020). Psychological interventions for people affected by the COVID-19 epidemic. Lancet Psychiatry.

[3] Holmes EA, O’Connor RC, Perry VH, Tracey I, Wessely S, Arseneault L, et al. Multidisciplinary research priorities for the COVID-19 pandemic: a call for action for mental health science. Lancet Psychiatry. 2020;15. 10.1016/S2215-0366(20)30168-1 PMC7159850 32304649

[4] Huang Y, Zhao N (2020). Generalized anxiety disorder, depressive symptoms and sleep quality during COVID-19 outbreak in China: a web-based cross-sectional survey. Psychiatry Res.

[5] Rajkumar RP (2020). COVID-19 and mental health: a review of the existing literature. Asian J Psychiatry.

[6] Cao J, Wei J, Zhu H, Duan Y, Geng W, Hong X (2020). A study of basic needs and psychological wellbeing of medical workers in the fever clinic of a tertiary general hospital in Beijing during the COVID-19 outbreak. Psychother Psychosom.

[7] Fagiolini A, Cuomo A, Frank E (2020). COVID-19 diary from a psychiatry department in Italy. J Clin Psychiatry.

[8] Gavin B, Hayden J, Adamis D, McNicholas F (2020). Caring for the psychological well-being of healthcare professionals in the Covid-19 pandemic crisis. Ir Med J.

[9] Hu X, Huang W. Protecting the psychological well-being of healthcare providers affected by the COVID-19 outbreak: implications for the psychological rescue work of international community. Nurs Health Sci. 2020;26. 10.1111/nhs.12727 PMC7267142 32335991

[10] Maher A, Rouprêt M, Misrai V, Pinar U, Matillon X, Tellier BG (2020). COVID-19 outbreak situation and its psychological impact among surgeon in training in France. World J Urol.

[11] Maben J, Bridges J. Covid-19: supporting nurses’ psychological and mental health. J Clin Nurs. 2020;22. 10.1111/jocn.15307 PMC7264545 32320509

[12] Lu W, Wang H, Lin Y, Li L (2020). Psychological status of medical workforce during the COVID-19 pandemic: a cross-sectional study. Psychiatry Res.

[13] Liu S, Yang L, Zhang C, Xiang Y-T, Liu Z, Hu S (2020). Online mental health services in China during the COVID-19 outbreak. Lancet Psychiatry.

[14] Chew NWS, Lee GKH, Tan BYQ, Jing M, Goh Y, Ngiam NJH, et al. A multinational, multicentre study on the psychological outcomes and associated physical symptoms amongst healthcare workers during COVID-19 outbreak. Brain Behav Immun. 2020;21:559–65. 10.1016/j.bbi.2020.04.049 PMC7172854 32330593

[15] Zhang C, Yang L, Liu S, Ma S, Wang Y, Cai Z (2020). Survey of insomnia and related social psychological factors among medical staff involved in the 2019 novel coronavirus disease outbreak. Front Psychiatry.

[16] Ullah R, Amin S (2020). The psychological impact of COVID-19 on medical students [letter]. Psychiatry Res.

[17] Maunder RG (2009). Was SARS a mental health catastrophe?. Gen Hosp Psychiatry.

[18] Kwek S-K, Chew W-M, Ong K-C, Ng AW-K, Lee LS-U, Kaw G (2006). Quality of life and psychological status in survivors of severe acute respiratory syndrome at 3 months postdischarge. J Psychosom Res.

[19] Maunder RG, Lancee WJ, Balderson KE, Bennett JP, Borgundvaag B, Evans S (2006). Long-term psychological and occupational effects of providing hospital healthcare during SARS outbreak. Emerg Infect Dis.

[20] Lancee WJ, Maunder RG, Goldbloom DS (2008). Prevalence of psychiatric disorders among Toronto hospital workers one to two years after the SARS outbreak. Psychiatr Serv Wash DC.

[21] Chen Q, Liang M, Li Y, Guo J, Fei D, Wang L (2020). Mental health care for medical staff in China during the COVID-19 outbreak. Lancet Psychiatry.

[22] Amanullah S, McNally K, Zelin J, Cole J, Cernovsky Z (2017). Are burnout prevention programs for hospital physicians needed?. Asian J Psychiatry.

[23] Gartlehner G, Forneris CA, Brownley KA, Gaynes BN, Sonis J, Coker-Schwimmer E (2013). Interventions for the prevention of posttraumatic stress disorder (PTSD) in adults after exposure to psychological trauma.

[24] Cheng P, Kalmbach DA, Tallent G, Joseph CL, Espie CA, Drake CL. Depression prevention via digital cognitive behavioral therapy for insomnia: a randomized controlled trial. Sleep. 2019;42(10). 10.1093/sleep/zsz150 PMC6783888 31535688

[25] Rose RD, Buckey JC, Zbozinek TD, Motivala SJ, Glenn DE, Cartreine JA (2013). A randomized controlled trial of a self-guided, multimedia, stress management and resilience training program. Behav Res Ther.

[26] Smoktunowicz E, Lesnierowska M, Cieslak R, Carlbring P, Andersson G (2019). Efficacy of an Internet-based intervention for job stress and burnout among medical professionals: study protocol for a randomized controlled trial. Trials..

[27] Joyce S, Shand F, Tighe J, Laurent SJ, Bryant RA, Harvey SB. Road to resilience: a systematic review and meta-analysis of resilience training programmes and interventions. BMJ Open. 2018;8(6). 10.1136/bmjopen-2017-017858 PMC6009510 29903782

[28] Joyce S, Shand F, Bryant RA, Lal TJ, Harvey SB. Mindfulness-based resilience training in the workplace: pilot study of the internet-based Resilience@Work (RAW) Mindfulness Program. J Med Internet Res. 2018;20(9) Available from: https://www.ncbi.nlm.nih.gov/pmc/articles/PMC6231729/. [cited 2020 Apr 13]. 10.2196/10326 PMC6231729 30206055

[29] Ruwaard J, Lange A, Broeksteeg J, Renteria-Agirre A, Schrieken B, Dolan CV (2013). Online cognitive-behavioural treatment of bulimic symptoms: a randomized controlled trial. Clin Psychol Psychother.

[30] Cohen S, Kamarck T, Mermelstein R (1983). A global measure of perceived stress. J Health Soc Behav.

[31] Kroenke K, Spitzer RL (2002). The PHQ-9: a new depression diagnostic and severity measure. Psychiatr Ann.

[32] Lesage F-X, Berjot S, Deschamps F (2012). Psychometric properties of the French versions of the Perceived Stress Scale. Int J Occup Med Environ Health.

[33] Bellinghausen L, Collange J, Botella M, Emery J-L, Albert É. Factorial validation of the french scale for perceived stress in the workplace. Santé Publique. 2009;21(4):365–73. 20101815

[34] Arroll B, Goodyear-Smith F, Crengle S, Gunn J, Kerse N, Fishman T (2010). Validation of PHQ-2 and PHQ-9 to screen for major depression in the primary care population. Ann Fam Med.

[35] Zuromski KL, Ustun B, Hwang I, Keane TM, Marx BP, Stein MB (2019). Developing an optimal short-form of the PTSD Checklist for DSM-5 (PCL-5). Depress Anxiety.

[36] Vaishnavi S, Connor K, Davidson JRT (2007). An abbreviated version of the Connor-Davidson Resilience Scale (CD-RISC), the CD-RISC2: psychometric properties and applications in psychopharmacological trials. Psychiatry Res.

[37] Bastien CH, Vallières A, Morin CM (2001). Validation of the insomnia severity index as an outcome measure for insomnia research. Sleep Med.

[38] Cropley M, Michalianou G, Pravettoni G, Millward LJ (2012). The relation of post-work ruminative thinking with eating behaviour. Stress Health J Int Soc Investig Stress.

[39] Devilly GJ, Borkovec TD (2000). Psychometric properties of the credibility/expectancy questionnaire. J Behav Ther Exp Psychiatry.

[40] Attkisson CC, Zwick R (1982). The Client Satisfaction Questionnaire: psychometric properties and correlations with service utilization and psychotherapy outcome. Eval Prog Plann.

[41] Richards D, Richardson T (2012). Computer-based psychological treatments for depression: a systematic review and meta-analysis. Clin Psychol Rev.

[42] Xiao H, Zhang Y, Kong D, Li S, Yang N (2020). The effects of social support on sleep quality of medical staff treating patients with coronavirus disease 2019 (COVID-19) in January and February 2020 in China. Med Sci Monit Int Med J Exp Clin Res.

[43] Folkman S, Lazarus RS. Stress, Appraisal, and Coping. New York: Springer Publishing Company; 1984. p. 460.

[44] Hayes SC, Luoma JB, Bond FW, Masuda A, Lillis J (2006). Acceptance and commitment therapy: model, processes and outcomes. Behav Res Ther.

[45] Gilbert P (2014). Compassion-focused therapy: preface and introduction for special section. Br J Clin Psychol.

[46] Neff KD, Germer CK (2013). A pilot study and randomized controlled trial of the mindful self-compassion program. J Clin Psychol.

